# Current trends of unicompartmental knee arthroplasty (UKA): choosing between robotic-assisted and conventional surgeries and timing of procedures

**DOI:** 10.1186/s42836-024-00289-5

**Published:** 2025-02-03

**Authors:** Kelvin S. C. Cheung, Kai Chun Augustine Chan, Amy Cheung, Ping Keung Chan, Michelle Hilda Luk, Kwong Yuen Chiu, Henry Fu

**Affiliations:** 1 Department of Orthopaedics and Traumatology, The University of Hong Kong, Hong Kong SAR, China; 2 Department of Orthopaedics and Traumatology, Queen Mary Hospital, Hong Kong SAR, China; 3 Department of Orthopaedics and Traumatology, Hong Kong Sanatorium Hospital, Hong Kong SAR, China

**Keywords:** Robotic surgery, Unicompartmental knee arthroplasty, Mako, Navio, Knee surgery, Osteoarthritis

## Abstract

**Background:**

With robotic advancements in UKA technology, we sought to explore if robotic-assisted UKA could translate to clinical benefits such as reduced hospital stays and lowered emergency readmissions. Also, current utilization trends of UKA and choice of procedure timing (unilateral [uUKA] vs. one-staged bilateral UKA [biUKA]) could be explored.

**Methods:**

This was a retrospective study utilizing the Clinical Data Analysis and Reporting System (CDARS) for data retrieval. All patients who had undergone primary UKA in all Hospital Authority (HA) hospitals in HK from 2021–2023 were included. Primary outcomes included utilization of UKA compared to TKA and percentage utilization of different UKA systems, namely, conventional, Mako, and Cori/Navio systems, from 2021–2023. Secondary outcomes involved: (1) patient demographics, (2) postoperative average length of stay (ALOS), (3) 30-day and 90-day postoperative Accident and Emergency Department (AED) attendance, (4) surgical times (skin-to-skin) and (5) 90-day mortality and reoperation. Differences in outcomes between uUKA and biUKA and between different robotic systems were examined. Regression analysis was performed to study if utilization of robotic-assisted systems could contribute to reduced hospital stays.

**Results:**

UKA accounted for 15.2% of primary knee arthroplasties throughout 2021–2023. Robotic-assisted UKA (Mako and Navio/Cori) has shown an increasing utilization since 2022 in both unilateral (16.0% to 25.9%) and bilateral operations (17.8% to 29.0%). Mako had shorter ALOS than Navio/Cori (2.9 ± 1.6 vs. 3.6 ± 2.6 days; *P* = 0.006) and significantly shorter ALOS than conventional UKA (2.9 ± 1.6 vs. 3.6 ± 2.6 days; *P* = 0.004). Utilization of Mako was predictive of shortened ALOS on multi-linear regression (β = − 0.056; *P* = 0.049). Interestingly, biUKAs, especially conventional ones, showed a lower attendance rate than uUKAs at 30-day (2.9% VS 6.9%; *P* = 0.036) and 90-days (7.8% VS 15.7%; *P* = 0.004). Robotic-assisted surgery was associated with a prolonged surgical time of 16.4 min in uUKA and 29.1 min in biUKA compared to conventional operations.

**Conclusion:**

UKA utilization has dropped since 2021 but the percentage of robotic-assisted UKA has risen. Mako yielded promising results in reducing hospital stays compared to conventional operations. Sub-group analysis (Mako versus Cori/Navio) highlighted the importance of distinguishing between different robotic platforms. For patients with bilateral unicompartmental OA, biUKA was shown to be a safe and effective alternative to unilateral operations.

**Trial Registration:**

Registered (HKU/ HA HKW IRB; Ref No: 24–373).

## Introduction

Knee osteoarthritis (OA) has posed increasing burden on healthcare systems across the globe in recent years due to aging population [1, 2]. In 2019, about 528 million people worldwide were living with osteoarthritis with an increase of 113% since 1990. When conservative treatment to manage symptoms of knee osteoarthritis fails, common surgical options for knee OA includes total knee arthroplasty (TKA) and for patients with predominately medial compartment osteoarthritis of the knee, an unicompartmental knee arthroplasty (UKA) can be used [3]. Studies have shown that UKA, a relatively bone-preserving and ligament-sparing procedure, could result in a reduced hospital stay and surgical time, and cost-saving [4, 5]. Despite perceived advantages of UKA, concerns remain regarding its higher revision rates compared to TKA [6, 7]. Hence, UKAs are only performed in highly-selective cases and there has been debate on the optimal percentage utilization of UKA in healthcare systems considering its relative advantages and drawbacks compared to the commonly performed TKA [8]. Despite higher revision rates, a converted UKA was shown to be as effective as primary TKA and superior to revision TKA [9].

With advancements in surgical technology, robotic assistance has been incorporated into UKA surgical workflow. Compared to conventional UKA, the robotic-assisted UKA aims to improve the accuracy of implant placement and limb alignment [10–13]. Sparked by better instrumentation and new robotic techniques, UKA has witnessed an increased utilization locally compared to 20 years ago [14]. In Hong Kong (HK), the first robotic-assisted UKA (Mako) was introduced in 2019. Currently in HK, the Mako and Navio/Cori systems are the most common robotic system used for UKA surgeries.

Despite promising results in surgical precision, robotic-assisted UKA systems also come with high initial costs for both the robot itself and maintenance [5, 15–17]. It is important to recognize whether healthcare investment in these systems could result in favorable clinical outcomes. Regardless of surgical systems used, debates continue about the utilization of UKA. For example, no consensus has been reached on the optimal usage of UKA in a public healthcare system [8, 10]. Also, clear indications on timing of procedure, and whether clinicians should opt for a staged procedure or one-stage operation, are not clear [18–20]. A comprehensive territory-wide review is warranted to find clearer recommendations on UKA utilization.

The aim of our study was to compare the utilization of UKA with TKA in HK and evaluate utilisation trends of robotic-assisted UKA systems with conventional UKA. Our study also sought to determine if UKA utilization rate has been optimal in recent years and whether robotic-assisted UKA could result in favorable outcomes such as reduced hospital stays and lowered AED attendance rate, which would, in turn, mitigate the burden on our healthcare system. In addition, differences in clinical outcomes between unilateral (uUKA) and one-staged bilateral UKA (biUKA), as well as different robotic systems were explored. Our results will inform clinicians on the choice of surgical system and timing of procedures when performing UKA.

## Methods

### Study design and patient selection

This was a retrospective study utilizing the Clinical Data Analysis and Reporting System (CDARS) for data retrieval. CDARS was created in the 1990s by the Hospital Authority of Hong Kong (HA) and has been serving as a local electronic medical database for clinical research. Clinical data in CDARS include patient demographics, diagnoses, operation records, admission/ discharge dates and patient mortality. Patient diagnoses were coded according to the International Classification of Diseases, Ninth revision (ICD-9) in CDARS. Ethics approval was obtained from local Institutional Review Board (IRB Ref: UW24-373).

All patients who had undergone primary UKA in all HA hospitals in HK between 2021 to 2023 were included in this study. Arthroplasties involving medial/lateral condyle and patellofemoral joint (PFJ) of the knee were included but bicompartmental arthroplasty was excluded. Patients were identified according to the procedural codes used in CDARS. The timeframe from 2021 to 2023 was used to identify patients who underwent robotic-assisted UKA operations since CDARS was only able to identify this group of patients after 2021. Included patient diagnoses were primary osteoarthritis (OA), secondary OA and osteonecrosis of the knee.

All patients underwent either conventional or robotic-assisted UKA. Those receiving robotic-assisted UKA were operated on by using either of the two robotic UKA systems, i.e., Mako and Navio/Cori. Conventional UKA is defined as operations using the traditional Oxford UKA surgical workflow. Oxford UKA involved use of a spherical articulating femoral component with a flat tibial component. Unconstrained high-density polyethylene bearing was inserted between the two components [21]. Mako UKA consisted of operations utilizing the Mako Robotic-arm Assisted System (Stryker, Mako Surgical Corp., Fort Lauderdale, FL, USA). It is the only 3D CT-image based robotic-arm-assisted surgical system and utilizes the Restoris MCK Implant System, which is composed of a femoral implant and a tibial baseplate. It allowed for preoperative implant planning for rotation, varus/valgus alignment, and degree of posterior slope [22]. Navio/Cori was defined as UKA using Navio/Cori surgical system (Smith & Nephew, INC., Memphis, TN, USA). In contrast to Mako, Navio/Cori employs an imageless guidance system and a high-speed handheld burr during surgical workflow. Currently, conventional Oxford UKA is commonly performed in 15 public hospitals across HK, while Mako and Navio/Cori are utilized in 10 and 8 hospitals respectively.

### Outcomes of interest

Primary outcomes of our study included utilization of UKA compared to TKA and percentage utilization of different UKA systems in HK from 2021–2023. Secondary outcomes included (1) patient demographics and operative diagnosis, (2) postoperative average length of stay (ALOS), (3) 30-day and 90-day Accident and Emergency Department (AED) attendance, (4) Surgical times (skin-to-skin) and (5) 90-day mortality and reoperation. Postoperative complication rate was assessed in terms of AED attendance and the corresponding diagnosis. Complications of interest included joint inflammation, minor wound infection, periprosthetic joint infection (PJI) and others unrelated orthopedic and medical problems. In addition, ALOS, AED attendance, and surgical times were compared between robotic subgroups (Mako vs. Cori/ Navio) and between uUKA and biUKA. Clinical outcomes like ALOS are multifactorial and contribute significantly to public healthcare expenditure. We assessed if the choice of surgical system, together with other patient factors, could contribute to improvement in ALOS.

### Data analysis

The independent-samples *t*-test and one-way ANOVA were used for comparison between continuous variables, and the *Chi*-square test for categorical variables. Odds ratio was calculated by *Chi*-square test. Multi-linear regression models were utilized to assess factors contributing to ALOS in UKA patients. All analyses were undertaken by using SPSS statistics (v29.0; IBM, USA). Two-tailed significance was set at *P* < 0.05.

## Results

### UKA utilization trend

**Fig. 1:**
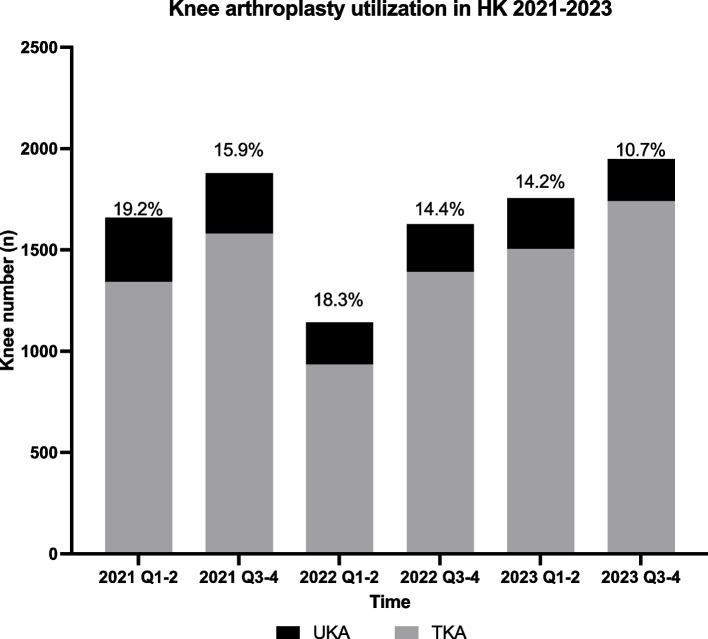
Utilization of UKA compared to TKA from 2021–2023

A total of 1255 patients involving 1519 knees met our inclusion criteria and were included. Overall, UKA constituted 15.2% (1519 out of 10,013) of primary knee arthroplasties throughout 2021–2023. However, the proportion of UKAs performed has dropped from 19.2% to 10.7% by the end of 2023. (Fig. 1) Within the 1255 UKA cases, 991 (78.9%) and 264 (21.0%) were unilateral and bilateral operations, respectively. Preoperatively, baseline comorbidities of patients (hypertension, chronic kidney disease [CKD], obesity, hyperlipidemia) between conventional and robotic-assisted systems were comparable. Patient and surgical details are presented in Table 1.

**Table 1:** Patient demographics and utilization rate of UKA 2021–2023

**Surgical system**		**Conventional**	**Robotic**		***P*-value**
**Patient Data**			**Mako**	**Cori/Navio**	
Female: male ratio		59:41	59:41	52:48	** < 0.001**^**†**^
Mean age (range)		69.1 (44–87)	68.6 (49–82)	68.9 (57–89)	0.664
Number of cases (*n*, %)	Unilateral (*n* = 991)	803 (81.0%)	139 (14.0%)	49 (4.9%)	
	Bilateral (*n* = 264)	205 (77.7%)	43 (16.3%)	16 (6.1%)	
Total number of involved compartments (*n*, %), Total = 1519		1213 (79.9%)	225 (14.8%)	81 (5.3%)	
Compartment involvement	Medial (*n*, %)	1211 (99.8%)	219 (97.3%)	78 (96.3%)	
	Lateral (*n*, %)	1 (0.1%)	6 (2.7%)	3 (3.7%)	
	Patellofemoral (n, %)	1 (0.1%	0 (0)	0 (0)	
Preoperative baseline comorbidities (*n*, %)					
Hypertension		208 (20.6)	32 (17.6)	10 (15.4)	0.410
Diabetes mellitus		115 (11.4)	10 (5.5)	4 (6.2)	0.029^**†**^
Hyperlipdemia		148 (14.7)	30 (16.5)	12 (18.5)	0.613
Obesity (BMI > 25)		406 (40.3)	70 (38.5)	25 (38.5)	0.126
Chronic kidney disease (CKD)		19 (1.9)	2 (1.1)	0 (0)	0.418
Operative diagnosis (*n*, % within group)					
Unilateral	Primary OA	795 (99.0%)	137 (98.6%)	48 (98.0%)	
	Secondary OA	1(0.1%)	1 (0.7%)	0 (0)	
	Osteonecrosis of knee	7 (0.9%)	1(0.7%)	1 (2.0%)	
Bilateral	Primary OA	204 (99.5%)	43 (100%)	16 (100%)	
	Secondary OA	0 (0)	0 (0)	0 (0)	
	Osteonecrosis of knee	1(0.5%)	0 (0)	0 (0)	

^†^Statistical significance

**Fig. 2:**
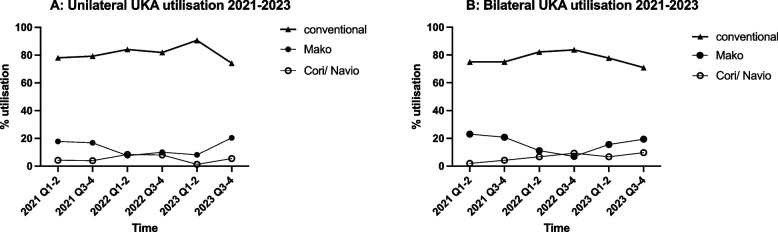
UKA utilization trend during 2021–2023 of different surgical subgroups: 2A and 2B show percentage UKA utilization in unilateral and bilateral operations respectively, with conventional UKA remaining the mainstay of treatment in both cases

Within UKA cases, conventional UKA remained the mainstay of treatment, consisting of 81.0% and 77.7% of unilateral and bilateral UKAs, respectively. The utilization rate of Mako and Navio/Cori was at 14.0% and 4.9% for unilateral UKAs, 16.3%, and 6.06% for bilateral ones, respectively. Overall robotic system utilization (Mako and Navio/Cori) has shown an increasing trend since 2022 in both unilateral (Fig. 2A: 16.0% to 25.9%) and bilateral operations (Fig. 2B: 17.8% to 29.0%).

### Postoperative Average Length of Stay (ALOS)

For uUKA, robotic-assisted UKAs had similar ALOS compared to conventional (3.5 ± 1.7 vs. 3.6 ± 2.6 days; *P* = 0.562). Subgroup analysis of robotic systems showed that Mako had shorter ALOS than Navio/Cori (2.9 ± 1.6 vs. 3.6 ± 2.6 days; *P* = 0.006) and was significantly lower than that of conventional systems (2.9 ± 1.6 vs. 3.6 ± 2.6 days; *P* = 0.004). For biUKA, conventional UKA had shorter ALOS than robotic-assisted UKA (3.9 ± 2.4 vs. 4.8 ± 2.7 days; *P* = 0.011). However, Mako biUKA showed no difference with conventional operation (4.3 ± 2.2 vs. 3.9 ± 2.4 days; *P* = 0.363) while ALOS was longer with Cori/Navio (6.4 ± 3.3 vs. 3.9 ± 2.4 days; *P* < 0.001). Intergroup comparison between Mako and Navio/Cori showed that Mako had shorter ALOS in both cases (*P* = 0.006). ALOS was similar between conventional uUKA and biUKA (*P* = 0.131). BiUKA had an ALOS 1.46 times that of Mako uUKA and 1.76 times that of Cori/Navio uUKA respectively (*P* < 0.001). (Table 2).

**Table 2:** Table showing average length of stay of UKA systems

**Surgical system**	**ALOS (days) ± SD**	**Difference (95% CI)**	*P*-value*
Unilateral			
Conventional	3.6 ± 2.6	Reference	Reference
Robotic	3.5 ± 1.7	− 0.1 (− 0.5 to 0.3)	0.562
Mako	2.9 ± 1.6	− 0.7 (− 0.3 to − 1.0)	**0.004**^**†**^
Cori/Navio	3.6 ± 1.4	+ 0.1 (− 0.2 to 0.5)	0.890
Bilateral			
Conventional	3.9 ± 2.4	Reference	Reference
Robotic	4.8 ± 2.7	+ 0.9 (0.2 to 1.7)	**0.011**^**†**^
Mako	4.3 ± 2.2	+ 0.4 (− 0.4 ± 1.1)	0.363
Cori/Navio	6.4 ± 3.3	+ 2.5 (1.2 to 3.8)	** < 0.001**^**†**^

^*^Comparisons made with reference group by independent samples *t*-test; ALOS, average length of stay; SD, standard deviation; CI, confidence interval; †Statistical significance

Multi-linear regression was conducted to assess variables affecting ALOS in UKA patients. BiUKA was found to be associated with an increase in ALOS, regardless of the surgical system used (β [standardized beta] = 0.106; *P* < 0.001). Assessment of individual robotic-assisted systems showed that use of Mako UKA was associated with a slight shortening of ALOS (β = − 0.056; *P* = 0.049). In contrast, ALOS was increased with the use of Navio UKA (β = 0.066; *P* = 0.029). Presence of CKD in patients was strongly associated with prolonged ALOS (β = 0.171; *P* < 0.001). Age, male gender, and other comorbidities were not associated with the length of hospital stay. (Table 3).

**Table 3:** Multiple linear regression models predicting prolonged length of stay in UKA patients

Model 1: Conventional UKA vs. robotic-assisted UKA		Dependent variable: ALOS				
Model Summary (number of patients analysed, *n* = 1255)		R	R^2^	R^2^ adjusted	Standard error of estimate	*P*-value
Independent variables		0.208	0.043	0.036	2.45	** < 0.001**^†^
		Unstandardized Beta	Standard error	Standardized β	*t*-test statistic	*P*-value
	Robotic-assisted UKA	− 0.106	0.175	− 0.017	− 0.602	0.547
	Laterality (Bilateral UKA)	0.650	0.171	0.106	3.791	**< 0.001**^†^
	Age	0.017	0.011	0.045	1.615	0.107
	Gender (male)	− 0.061	0.142	− 0.012	− 0.433	0.665
	Hypertension	0.142	0.191	0.023	0.742	0.458
	Diabetes mellitus	− 0.053	0.245	− 0.006	− 0.215	0.830
	CKD	3.326	0.545	0.171	6.101	**< 0.001**^†^
	Obesity (BMI > 25)	− 0.119	0.143	− 0.023	− 0.833	0.405
	Hyperlipidemia	0.064	0.212	0.009	0.301	0.764
Model 2: Conventional UKA vs. Mako UKA		Dependent variable: ALOS				
Model Summary (*n* = 1190)		R	R^2^	R^2^ adjusted	Standard error of estimate	*P*-value
Independent variables		0.210	0.044	0.037	2.46	**< 0.001**^†^
		Unstandardised Beta	Standard error	Standardised β	*t*-test statistic	*P*-value
	Mako UKA	− 0.392	0.199	− 0.056	− 1.973	**0.049**^†^
	Bilateral UKA	0.513	0.177	0.083	2.898	**0.004**^†^
	Age	0.016	0.011	0.041	1.429	0.153
	Gender (male)	− 0.025	0.146	− 0.005	− 0.171	0.864
	Hypertension	0.110	0.196	0.018	0.564	0.573
	Diabetes mellitus	− 0.028	0.250	− 0.003	− 0.112	0.911
	CKD	3.341	0.546	0.176	6.114	**< 0.001**^†^
	Obesity (BMI > 25)	− 0.165	0.147	− 0.032	− 1.128	0.260
	Hyperlipidemia	0.073	0.219	0.010	0.336	0.737
Model 3: Conventional UKA vs. Navio/Cori UKA		Dependent variable: ALOS				
Model Summary (*n* = 1073)		R	R^2^	R^2^ adjusted	Standard error of estimate	*P*-value
Independent variables		0.215	0.046	0.038	2.54	**< 0.001**^†^
		Unstandardized Beta	Standard error	Standardized β	*t*-test statistic	*P*-value
	Navio/Cori UKA	0.715	0.326	0.066	2.190	**0.029**^†^
	Bilateral UKA	0.519	0.193	0.081	2.688	**0.007**^†^
	Age	0.016	0.012	0.040	1.319	0.188
	Hypertension	0.138	0.211	0.021	0.654	0.514
	Diabetes mellitus	0.034	0.265	− 0.004	− 0.126	0.899
	CKD	3.594	0.595	0.183	6.040	**< 0.001**^†^
	Obesity	− 0.151	0.159	− 0.029	− 0.946	0.344
	Hyperlipidemia	0.126	0.237	0.017	0.531	0.596

ALOS, Average length of stay; R, coefficient of correlation; R^2^, coefficient of determination; CKD, chronic kidney disease; BMI, body-mass index; †Statistical significance

### AED attendance rate

**Fig. 3:**
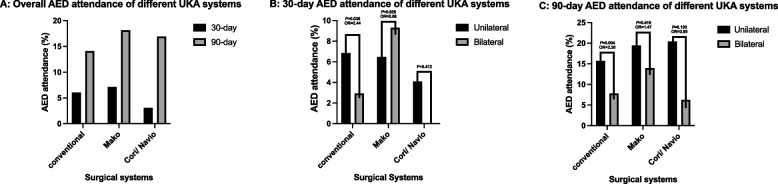
Bar chart showing AED attendance rates of different UKA systems: 3A, No difference was observed between different UKA systems in AED attendance rates; 3B, 30-day AED attendance rates were significantly lower in conventional biUKA than in uUKA; 3C, 90-day AED attendance rates of all biUKA were lower than those of uUKA across all systems, with conventional UKA being most significant

At 30 days, overall attendance rate for conventional UKA was at 6.05% and was not significantly different from that with Mako (6.1% vs. 7.1%; *P* = 0.58) and Navio/Cori (6.1% vs. 3.1%; *P* = 0.323). The rate of minor postoperative wound infection was higher in Navio/Cori, being at 1.5% (One-way ANOVA; *P* < 0.001). (Table 4) Conventional biUKA was associated with a lower 30-day attendance rate compared with uUKA (2.9% vs. 6.9%; Odds ratio (OR) = 0.410; *P* = 0.036) while there was no statistical difference noted with robotic systems. (Fig. 3).

**Table 4:** Diagnoses of patients attending AED at 30-days

**Surgical systems**		**Attendance number (*n*, %)**	**Postoperative joint inflammation (*n*, %)**	**Minor postoperative wound infection (*n*, %)**	**Prosthetic joint infection (*n* (%)**	**Unrelated orthopedic diagnoses (*n*, %)**	**Unrelated medical diagnoses (*n*, %)**
Conventional		61(6.1)	1(0.1)	0(0)	0(0)	2(0.2)	58(5.8)
Robotic	Mako	13(7.1)	0(0)	0(0)	0(0)	2(1.1)	11(6.0)
	Navio/Cori	2(3.1)	0(0)	1(1.5)	0(0)	1(1.5)	0(0)
*P*-value*			0.885	** < 0.001**^**†**^	/	0.065	0.144

^*^Comparisons made using One-way ANOVA; †Statistical significance

At 90 days, the overall AED attendance rate for Mako and Navio/Cori was 18.1 and 16.9%, respectively, with no statistical differences observed between the 2 robotic groups (*P* = 0.827) and with no increase in the rate compared to conventional operation (14.1 vs. 18.1% and 14.1 vs. S 16.9%; *P* = 0.156 and *P* = 0.526). (Fig. 3A) Similarly, conventional biUKA was associated with lower attendance rate than its unilateral counterpart (7.8% vs. 15.7%; OR = 0.455; *P* = 0.004). Although not statistically significant, both Mako biUKA (13.6% vs. 19.4%; OR = 0.673; *P* = 0.416) and Cori/Navio biUKA (6.3% vs. 20.4%; OR = 0.260; *P* = 0.190) had lower attendance rate compared to a patient who underwent unilateral UKA. (Fig. 3B and C) Minor wound infections were more common in Navio/Cori (1.5%) compared to the conventional UKA system (0.1%; One-way ANOVA; *P* = 0.016). There were no differences in postoperative joint inflammation (joint pain and knee effusion) and medical problems (cardiovascular, gastrointestinal, pulmonary) between all groups at both 30 and 90 days. No cases of prosthetic joint infection (PJI) were identified for up to 90 days in our study. (Table 5).

**Table 5:** Diagnoses of patients attending AED at 90-days

**Surgical systems**		**Attendance number (%)**	**Postoperative joint inflammation (*n*, %)**	**Minor postoperative wound infection (*n*, %)**	**Prosthetic joint infection (*n*, %)**	**Unrelated orthopedic diagnoses (*n*, %)**	**Unrelated medical diagnoses (*n*, %)**
Conventional		142 (14.1)	5 (0.5)	1 (0.10)	0 (0)	10 (0.1)	126 (12.5)
Robotic	Mako	22 (18.1)	1 (0.6)	0 (0)	0 (0)	4 (2.2)	28 (15.4)
	Navio/Cori	11 (16.9)	0 (0)	1 (1.5)	0 (0)	4 (6.2)	6 (9.2)
*P*-value *			0.844	**0.016**^**†**^	/	**0.002**^**†**^	0.384

^*^Comparisons made using One-way ANOVA; †Statistical significance

### Surgical time

Surgical time for unilateral UKA was shortest with conventional UKA system, being at 88.4 ± 25.0 min, lower than that of Navio/Cori (VS 101.1 ± 23.7 min; *P* < 0.001) and Mako (VS 106.2 ± 25.3 min; *P* < 0.001). Similar results were observed for bilateral operations, that is, conventional UKA had an average surgical time of 160.2 ± 34.8 min, which less compared to Mako (VS 190.2 ± 38.2 min; *P* < 0.001) and Navio/Cori (VS 186.8 ± 28.9 min; *P* = 0.003). No statistical difference was found between the 2 robotic systems in surgical time in uUKA (*P* = 0.215) and biUKA cases (*P* = 0.744). (Table 6).

**Table 6:** Table showing mean surgical times (skin-to-skin) of UKA systems

**Surgical system**	**Surgical times (mins) ± SD**	**Difference (95% CI)**	*P*-value*
**Unilateral**			
Conventional	88.4 ± 25.0	Reference	Reference
Robotic	104.9 ± 25.0	+ 16.5 (12.5 to 20.4)	** < 0.001**^**†**^
Mako	106.2 ± 25.3	+ 17.8 (13.3 to 22.3)	** < 0.001**^**†**^
Cori/Navio	101.0 ± 23.7	+ 12.7 (5.4 to 19.9)	** < 0.001**^**†**^
**Bilateral**			
Conventional	160.2 ± 34.8	Reference	Reference
Robotic	189.2 ± 35.7	+ 29.0 (18.9 to 39.2)	** < 0.001**^**†**^
Mako	190.2 ± 38.2	+ 30.0 (18.3 to 41.7)	** < 0.001**^**†**^
Cori/Navio	186.8 ± 28.9	+ 26.5 (8.9 to 44.1)	**0.003**^**†**^

^*^Comparisons made with reference group by independent samples *t*-test; SD, standard deviation; CI, confidence interval; †Statistical significance

### Reoperation and mortality

No 90-day reoperation and mortality were recorded across all groups in 2021–2023.

## Discussion

In this study, we demonstrated a decreasing trend in UKA utilization rate during 2021–2023. For patients who underwent UKA, robotic-assisted UKA was found to be on the rise in recent years. No significant differences in AED attendance were observed between robotic-assisted UKA and conventional UKA. Subgroup analysis revealed that Mako UKA had a reduced hospital stay of 0.6 days compared to conventional UKA in unilateral cases, which was confirmed with our multi-linear regression model. Comparison of different robotic systems, Mako vs. Cori/Navio, showed difference in reduced hospital stay. Our data suggested that biUKA is a safe and effective alternative for the treatment of bilateral medial OA, given its benefit in reducing overall hospital stay and postoperative AED attendance.

Previously, local studies have highlighted that UKA utilization increased from 2002 to 2017 [14]. However, our study observed that the increasing trend did not continue in recent years, with the UKA utilization gradually dropping from 19.2% at the start of 2021 to 10.7% at the end of 2023 of all knee arthroplasties. The decrease in UKA utilization could be multifactorial. Indication for UKA demands early diagnosis of isolated unicompartmental OA involvement before the bicompartmental disease progression. Locally, waiting time for knee arthroplasties and the first assessment have been increasing significantly in recent years due to growing incidence of knee osteoarthritis owing to the aging local population [23, 24]. Hence, most patients presenting to local institutions have more advanced diseases and are symptomatic at presentation. In addition, the recent COVID pandemic has caused significant delays in elective surgeries, such as joint arthroplasties, further increasing the waiting time for these patients [25], and this could translate to disease progression beyond the indication for UKA. Compared to other countries, the National Joint Registry of the United Kingdom reported a steady UKA utilization rate of 13% over the past 2 years [26], while New Zealand and Australian Joint registry registered a reduced utilization rate of UKA, from 10.3% in 2022 to 7.3% 2023 [27, 28]. Globally, UKA utilization of UKA varies, but most were found to be below 20%. Previous literature has found that the optimal usage of UKA to be at 40–60%, while an acceptable UKA revision rate was achieved at > 20% and < 5% [8, 29]. The perceived advantages of UKA include less invasive surgery with more bone preservation and ligament sparing, reduced hospital stay, blood loss, and complication rate compared to TKA. Furthermore, revision of UKA to TKR following eventual failure of arthroplasty is less complicated than total knee arthroplasty revision [9]. It was proposed that clinicians should consider increasing UKA utilization to achieve desirable effect that UKA was originally designed for. Our data have suggested that there is trend of reduced utilization of UKA, which prompts further evaluation of the cause of relative underutilization of UKA in Hong Kong.

Within UKA patient groups, we observed an increased utilization of robotic-assisted surgeries. The increase in robotic-assisted UKA could be attributed to advantages provided by robotic systems, including more accurate alignment [12] and detailed preoperative planning as well as increased availability of the system to hospital authorities. Currently, four public hospitals in Hong Kong owned robotic knee systems while 10 public hospitals have robots on loan, totaling 14 public hospitals capable of performing robotic-assisted knee arthroplasty surgeries. A more widespread adoption of robotic-assisted UKA systems could be hindered by the high initial purchase cost of robots and, need for maintenance and training for surgeons.

Length of stay was neither increased nor reduced with robotic systems in comparison to conventional UKA, except for Mako. Previous studies have shown that Mako-assisted UKA had a shorter time to discharge compared to conventional operation [30–33], which was consistent with our results, suggesting that the Mako system results in significantly reduced postoperative stay in uUKA. Patients who underwent Mako UKA had reduced ALOS compared to Cori/ Navio UKA. Our regression mode showed that, together with CKD and laterality, use of Mako UKA was associated with shorter hospital stay in our regression model, but not with the Navio/Cori system. Kayani et al. suggested that intraoperative bone resection and soft tissue release were reduced with a CT-based robotic system for knee arthroplasty compared to conventional methods [34]. The reduced tissue trauma may contribute to less immediate periarticular inflammation, postoperative pain, quicker initiation of rehabilitation and, ultimately, quicker hospital discharge. Since Cori/Navio system adopts a different method of navigation, our data suggest that one should not treat all robotic systems equally and individual robotic navigation systems were found to yield different clinical outcomes. Mako is the only CT-based robotic system with surgery tailored to patient’s anatomy. The use of Mako robotic arm-mounted irrigated burr instead of conventional high-speed burr in Cori/Navio might minimize unnecessary bone removal and heat-associated bone necrosis, leading to reduced blood loss and lower postoperative pain scores [35]. Increased periarticular trauma in Navio/Cori surgeries could potentially explain its association with longer ALOS, given its imageless nature and use of high-speed burr. However, it should be noted the sample size of Navio/Cori patients was relatively small. Further research is required before we could conclude that one robotic system is superior to the other in reducing hospital stay. Reduction in hospital stay as demonstrated by Mako UKA is of particular importance in an Asian locality, since Asian varus knees are predisposed to medial compartment OA [36]. Locally, the main culprits for protracted ALOS include lack of social support following discharge, poor socioeconomic status, and suboptimal living environment of patients in the public sector. These factors significantly lower patients’ incentive for early discharge [37]. Physically, robotic-assisted UKA systems, especially Mako, could reduce postoperative inflammation, allowing for early rehabilitation. Psychologically, patients may perceive they recover quicker and would adopt a more accepting attitude to early discharge [38]. These findings could potentially contribute to reductions in in-hospital expenses in Hong Kong, which has been burdened by OA patients increasingly.

Both conventional and robotic-assisted UKAs exhibited similar AED attendance. Number of patients requiring further attention for joint inflammatory issues, such as effusion or swelling, were found to be similar across all groups. A systematic review performed by Bensa et al. reported lower postoperative complication rate with robotic-assisted UKA compared to conventional UKA (5.2% versus 10.1%) but the difference was not statistically significant [39]. Zhang et al. demonstrated similar total complication rates between conventional and robotic-assisted UKA [40]. Interestingly, Navio/Cori system showed more wound complications at 90-days (1.54% vs. 0% in Mako and 0.1% in conventional surgery). Mako allows for preoperative CT-based planning but Cori/Navio only allows for intraoperative registration and utilizes high-speed burring for bone cuts. The latter may result in inaccuracies during registration due to the lack of CT-based image reference, especially for the posterior tibial plateau. As aforementioned, inaccurate planning may cause more unnecessary intraoperative trauma, which could attribute to more postoperative problems and complications. Overall, robotic-assisted UKA was shown to be a safe alternative to conventional UKA as complications and emergency readmissions were not increased.

Robotic-assisted surgeries were associated with a longer surgical time of +16.4 min in uUKA and +29.1 min in biUKA compared to conventional UKA. This increase in surgical time was consistent with previous literature [39]. Longer surgical time in robotics could be attributed to time required for setting-up of the robot and registration with robotic system. Although longer surgical time may predispose patients to greater risk of surgical infection [41], the increase in complications was not seen in our study. Graham et al. reported that total operating time with robotic-assisted UKA was 34 min longer than conventional ones (133 vs. 99 min; *P* < 0.001), increasing total perioperative personnel costs ($1675 USD vs. $1218 USD in conventional UKA; *P* < 0.001) [16]. However, total costs were lower in robotic operations as it was shown that money saved from shorter hospital stay and implant choice could offset the cost increase resulting from longer surgical time [16, 31, 33]. Especially in high-volume hospitals where robotic-assisted UKA was highly utilized, utilization of robots was shown to be economically sustainable given its benefits in reducing revision rates and acute readmissions [15, 17]. As personnel costs and costs for hospital stay may vary across regions, further cost-effectiveness analysis should be done to ascertain the economic benefits that robotics could bring in our local setting.

Interestingly, our study results showed advantages of biUKA over uUKA in terms of ALOS as well as AED attendance rate at 90-days. ALOS in conventional biUKA was found to be similar to that in uUKA. For robotic-assisted UKA, the ALOS for biUKA was 1.40 times that of uUKA (4.83 vs. 3.46 days), suggesting biUKA results in a decreased ALOS of 2.1 days compared to staged UKA consisting of two unilateral surgeries. Furthermore, AED attendance rate at 90-days was lower in biUKA group, which is more significant with conventional biUKA. BiUKA has previously been shown to be more cost-effective in terms of reduced hospital stay, which could result in lower risk of postoperative complications, such as deep vein thrombosis and pulmonary embolism. Additional cost is required for two separate hospital stays in staged biUKA (*i.e*., two unilateral operations) in comparison to one single hospital stay in biUKA [18–20]. BiUKA has also been shown to have lowered postoperative complications compared to staged operations [20]. However, the longer waiting time for joint arthroplasty could result in deteroriation of patient condition, resulting in contraindication for UKA in patients otherwise suitable for UKA. Our data indicated that increasing utilization of biUKA could be used as a measure to reduce hospital stay and emergency readmissions, ultimately saving public healthcare costs. It should be noted that patients selected for biUKA were usually more fit for surgery, which could potentially be factored into the better clinical outcomes post-bilateral surgery. In patients with bilateral unicompartmental OA, biUKA was shown to be a safe and effective alternative.

This is the first territory-wide study to compare conventional and robotic-assisted UKA in Asia. However, our study does have some limitations. First, big data analysis utilizing CDARS was adopted in our study. We could not adjust for some confounding factors, such as surgeon and hospital factors. Further research comparing robotic-assisted and conventional UKA in a more controlled environment is warranted, such as large-scale randomized controlled trials to minimize the effects of confounders. Second, our study could not include patient-reported outcome measures (PROMs) as these data were not collected and stored in CDARS. Hence, we could not evaluate the outcomes of UKA from patients’ perspective, which is important in holistic care. Last, the size of our study population, especially the robotic-assisted UKA group, was relatively small. Given that there is the difference in number of patients between conventional and robotic-assisted groups, caution should be excised when interpreting the results of inter-group comparisons.

## Conclusion

UKA utilization has been on a decline since 2021, but the percentage of robotic-assisted UKA has risen. Public healthcare systems could consider the possibility of increasing UKA utilization to maximize the perceived benefits of the procedure. Comparing robotic to conventional UKA in general, no significant advantages were observed in terms of AED attendance. Sub-group analysis has revealed that Mako robotic-assisted surgery could reduce hospital stay following unilateral knee arthroplasty, which is of particular importance in an Asian locality. Comparison of robotic systems (Mako versus Cori/Navio) highlighted the importance of distinguishing between different robotic platforms and the method of navigation used could result in differences in clinical outcomes. For patients with bilateral unicompartmental OA, bilateral UKA is safe and effective since biUKA could effectively reduce overall hospital stay and postoperative emergency readmissions as compared to staged operations, thereby leading to reduced healthcare cost and socioeconomic burden posed by knee osteoarthritis.

## Data Availability

The datasets used and/or analyzed during the current study are not available to the public but could be made available from the corresponding author on reasonable request.

## Abbreviations

UKA: Unicompartmental knee arthroplasty
CDARS: Clinical Data Analysis and Reporting System
HK: Hong Kong
HA: Hospital Authority of Hong Kong
biUKA: Bilateral one-staged UKA
uUKA: Unilateral UKA
OA: Osteoarthritis
TKA: Total knee arthroplasty
AED: Accident & Emergency Department
SD: Standard deviation
CI: Confidence interval
ALOS: Average length of stay
ICD-9: International Classification of Diseases, Ninth revision
PJI: Prosthetic Joint Infection
CKD: Chronic kidney disease
